# Triacylglycerides and Phospholipids from Egg Yolk Differently Influence the Immunostimulating Properties of Egg White Proteins

**DOI:** 10.3390/nu13103301

**Published:** 2021-09-22

**Authors:** Leticia Pérez-Rodríguez, Mónica Martínez-Blanco, Daniel Lozano-Ojalvo, Javier Fontecha, Elena Molina, Sara Benedé, Rosina López-Fandiño

**Affiliations:** 1 Instituto de Investigación en Ciencias de la Alimentación (CIAL, CSIC-UAM), Nicolás Cabrera 9, 28049 Madrid, Spain; leticia.p.r@csic.es (L.P.-R.); m.martinez.blanco@csic.es (M.M.-B.); j.fontecha@csic.es (J.F.); e.molina@csic.es (E.M.); s.benede@csic.es (S.B.); 2 Icahn School of Medicine at Mount Sinai, 1425 Madison Ave, 11-26, New York, NY 10029, USA; daniel.lozano-ojalvo@mssm.edu

**Keywords:** food allergy, egg yolk, triacylglycerides, phospholipids, Th2 responses, group 2 innate lymphoid cells, dendritic cells

## Abstract

As part of a whole egg, egg white proteins are embedded in a lipid matrix that could modify their presentation to the immune system and their allergenic properties. The present study examines the impact of the main egg lipid components, triacylglycerides and phospholipids, in the early events of sensitization to egg. To this end, BALB/c mice were exposed intragastrically to egg lipids and egg lipid fractions, alone and in mixtures with egg white proteins, and Th2-promoting and proinflammatory effects were investigated. Our results highlight that the egg lipid fraction is responsible for Th2 adjuvant effects and point at a different influence of triacylglycerides and phospholipids on the bioavailability and immunomodulating properties of egg white proteins. While triacylglycerides promote type 2 responses at the small intestine level, phospholipids reduce the solubility of EW proteins and induce Th2 skewing in lymphoid intestinal tissues, which may have a direct impact on the development of egg allergy.

## 1. Introduction

Egg allergy is one of the most common food allergies in European children, with a prevalence that ranges between 2 and 7% depending on whether it is estimated on the basis of objective or self-reported assessments [1]. The evidence that the cases of egg allergy are growing is of great concern as a public health problem, because of its economic impact and negative effects on quality of life [2,3], inasmuch as allergy to egg frequently causes severe anaphylaxis and is a marker of later sensitization to aeroallergens and development of asthma [4]. Numerous studies have addressed the structural properties of egg white proteins (mainly ovalbumin, ovomucoid, and lysozyme) that determine their ability to sensitize and elicit allergic reactions, essentially associated with their high resistance to gastroduodenal digestion, which allows maintaining the integrity of their epitopes to induce Th2 cell differentiation and IgE-mediated activation of effector cells, as well as the effects of processing, particularly heat treatment (for a review see [5]). However, it is often ignored that, as part of a whole egg, egg white proteins are immersed in a lipid matrix that could affect their digestibility, gastrointestinal absorption, cellular recognition, and presentation to the immune system [6].

Previous studies, using mouse models of orally induced allergy, indicated that egg yolk provides Th2 adjuvant stimuli to the immune system that may enhance sensitization and susceptibility to develop egg allergy, an aspect also corroborated with other nonrelated food allergens, such as whey proteins [7]. The investigation of the mechanisms governing polarization towards a Th2 response, concentrating on the early intestinal immune and inflammatory effects in a murine model of acute feeding, showed that egg yolk may promote sensitization to egg white proteins through the activation of innate immune cells, such as intestinal epithelial cells (IECs), dendritic cells (DCs), and group 2 innate lymphoid cells (ILC2s) [8]. Communication among these cells, with IECs releasing cytokines, such IL-33, IL-25, and TSLP, that instruct DCs to promote Th2 responses and to stimulate the expansion of ILC2s (an extra source of polarizing cytokines, such as IL-4, IL-5, and IL-13), induces and reinforces Th2 immunity, which is critical in allergic sensitization [9,10,11,12].

The purpose of the present study was to determine the impact of egg lipids and their main components: triacylglycerides (representing approximately 60% of egg lipids) and phospholipids (30% of egg lipids) in sensitization to egg. To this end, BALB/c mice were administered egg white, as well as egg lipids and egg lipid fractions, alone and in mixtures with EW, for 6 consecutive days. Special attention was paid to gastrointestinal responses to the acute feeding regime by investigating the expression of genes encoding markers for barrier function, epithelial Th2-promoting cytokines, Th2-skewing factors of DCs, and effector Th2 and Th17 cell differentiation, both in nonlymphoid and lymphoid tissues. The populations of ILC2s and Th2 cells were also assessed, respectively, in the lamina propria and mesenteric lymph nodes of mice.

## 2. Materials and Methods

### 2.1. Samples

Fresh hen eggs, purchased at a local supermarket, were from the same production batch (No C.E: ES14.002873/z). Egg white and yolk were carefully separated from fresh eggs [7] and the resulting fractions (termed respectively EW and EY) were lyophilized. Lipid fractions were obtained as described [13]. Briefly, EY was extracted with 95% ethanol (10 mL g^−1^) with magnetic stirring at 65 °C for 1 h. The ethanol-soluble portion containing egg lipids was filtered and either lyophilized after removal of ethanol and used as the total egg lipid fraction (EL) or crystallized at 4 °C for 16 h. Following crystallization, solidified triacylglycerides and nonsolidified phospholipids were separated by centrifugation at 2800× *g* for 20 min. Ethanol was removed and samples (termed respectively TG and PL) were lyophilized. The analysis of lipid class composition by HPLC-ELSD [14] showed that the TG fraction consisted of 99.8% triacylglycerides, while the PL fraction was composed of 77.8% phospholipids (of which 83.2% was phosphatidylcholine and 16.4% phosphatidylethanolamine), 11.7% cholesterol, and 6.5% triacylglycerides (Supplementary Figure S1), which is in agreement with the results reported by Su et al. [13].

### 2.2. Experiments in Mice

**Figure 1:**
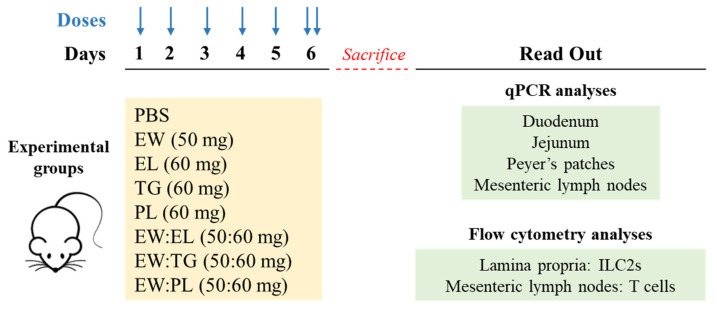
Experimental protocol in vivo. Mice were administered PBS, egg white (EW, 50 mg), egg lipids (EL, 60 mg), a triacylglyceride-enriched fraction (60 mg, TG), a phospholipid-enriched fraction (60 mg, PL), and their mixtures (50 mg:60 mg) with EW (respectively EW:EL, EW:TG, EW:PL) for 6 consecutive days.

Six-week-old female BALB/c mice were purchased from Charles River Laboratories (Saint Germain sur l’Arbresle, France). Mice (6 per group) were orally exposed to 0.2 mL of PBS; EW (50 mg); EL (60 mg); TG (60 mg); PL (60 mg); or the mixtures EW:EL, EW:TG, and EW:PL in a proportion 50 mg:60 mg, for six consecutive days (Figure 1). On the last day, mice received a boost administration 2 h after the first dose and were euthanized by CO_2_ inhalation 2 h later. The proportion of EW:EL administered to mice mimicked the ratio in hen eggs: on the basis of dry matter, hen eggs are composed of EW and EY in an approximate proportion of 1:2, with EL amounting for nearly 60% of the dry matter of EY.

Duodenum, jejunum, Peyer’s patches (PPs), and mesenteric lymph nodes (MLNs) were individually recovered for gene expression analyses. ILC2s from lamina propria and T cells from MLNs were analyzed by flow cytometry.

All protocols followed the European legislation (Directive 2010/63/EU) and were approved by Comunidad de Madrid (Ref PROEX 286.8/20).

### 2.3. Isolation of Cells from Lamina Propria

In order to obtain a single lamina propria cell suspension, the small intestine was collected, flushed, and washed three times with Hanks’ Balanced Salt Solution (HBSS, Corning Inc., Nueva York, NY, USA) containing 2% FBS. PPs were excised, and mucus and IECs were removed by incubation with 0.5 DTT and 1.3 mM EDTA (all from Biowest SAS, Nuaillé, France), respectively. The remaining segments were washed and treated with 1.6 mg mL^−1^ collagenase D and 10 U mL^−1^ DNase (both from Roche Pharma, Basel, Switzerland) for 40 min at 37 °C and 250 rpm, followed by separation on a discontinuous Percoll gradient (GE Healthcare, Uppsala, Sweden).

### 2.4. Gene Expression

Total RNA was isolated using RNA Kit (Macherey-Nagel Gmbh & Co., Düren, Germany) and reverse transcribed into cDNA using PrimeScript RT Reagent Kit (Takara Bio Inc., Shiga, Japan). Primer pairs and thermal cycling conditions for q-PCR assays were performed as described [8]. Relative gene expression was calculated by normalizing data to the expression of the *Actb* gene (encoding for β-actin) using the 2^−ΔΔCT^ method.

### 2.5. Flow Cytometry Analyses

Lamina propria and MLN cells were recovered in PBS containing 2% FBS and 1 mM EDTA. Fc receptors were blocked using anti-CD16/CD32 (clone 2.4G2, BD Biosciences, San Diego, CA, USA) and live cells were determined with LIVE/DEAD Fixable Near-IR Dead Cell Stain Kit (Thermo Fisher Scientific). Samples were stained with the antibodies listed in Supplementary Table S1. Cells were acquired with a Gallios flow cytometer (Beckman Coulter, Krefeld, Germany) and analyses were performed with FlowJo software (TreeStar Inc., San Carlos, CA, USA).

### 2.6. Solubility Experiments

The solubility of EW (5 mg) and its mixtures with EL, TG, and PL (5 mg:6 mg) was examined in 1 mL of 20.3 mM Bis-Tris containing 7.6 mM CaCl_2_ and a mixture of sodium glycodeoxycholate and sodium taurocholate (Sigma–Aldrich, St. Louis, MO, USA) (6.0 mM of each salt) at pH 6.5 to simulate duodenal conditions typical of an intermediate state between a fasted and a fed state. After 2 h at 37 °C, solubility was determined by SDS-PAGE, following centrifugation (10,000 g, 20 °C, 10 min) and resuspension of the precipitate in 1 mL PBS [15].

### 2.7. Statistical Analyses

Results are presented as means ± SEM. Differences between a control and an experimental group were assessed by the unpaired two-tailed Student’s *t*-test, and differences among three or more groups were determined by one-way ANOVA followed by Tukey post hoc test, except for gene expression data, which were evaluated by Mann–Whitney U test. *p* < 0.05 was considered statistically significant. Statistical analyses were performed using GraphPad Prism v6 (GraphPad Software Inc., San Diego, CA, USA).

## 3. Results

### 3.1. Egg Yolk Triacylglycerides Promote Type 2 Responses at the Small Intestine Level

**Figure 2:**
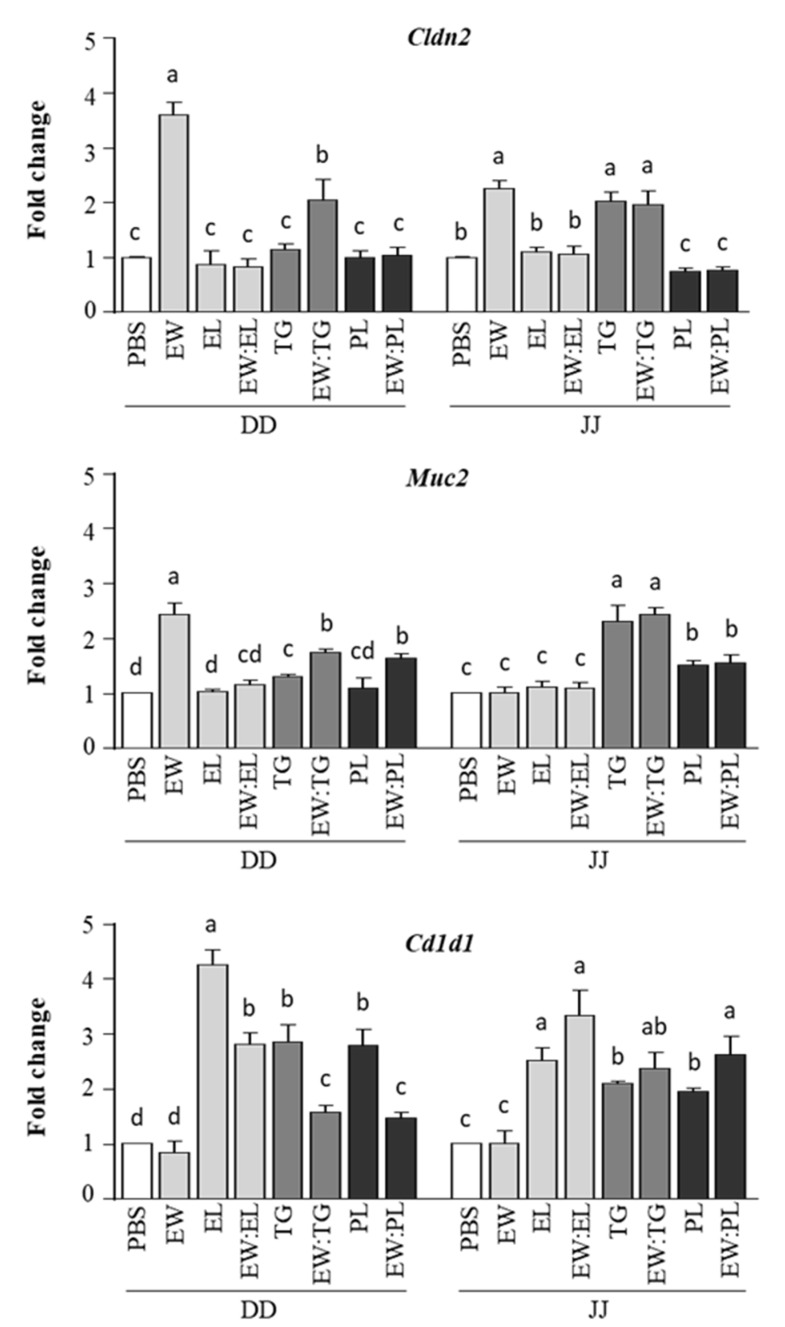
Relative gene expression of *Cln2, Muc2*, and *Cd1d1*, determined in the duodenum (DD) and jejunum (JJ) of mice intragastrically administered PBS, egg white (EW, 50 mg), egg lipids (EL, 60 mg), a triacylglyceride-enriched fraction (60 mg, TG), a phospholipid-enriched fraction (60 mg, PL), and their mixtures (50 mg:60 mg) with EW (respectively EW:EL, EW:TG, EW:PL) for 6 consecutive days. Gene expression was normalized to the reference gene *Actb* and compared with mice administered PBS. Data are expressed as means ± SEM (*n* = 6). Different letters indicate statistically significant differences (*p* < 0.05) among different mouse groups.

In agreement with earlier results [8], administration of EW to BALB/c mice enhanced the expression of *Cldn2* and *Muc2* in the duodenum and *Cldn2* in the jejunum (Figure 2). *Cldn2* and *Muc2* code, respectively, for Claudin-2, a pore-forming tight-junction protein [16], and Muc2, the major mucus constituent [17], and their upregulation by EW may be linked to a disruptive effect on intestinal barrier integrity that could favor its permeability and subsequent recognition by the immune system [8]. EL did not modify *Cldn2* or *Muc2* expression, and its administration together with EW abolished the effects of the latter on the regulation of these genes (Figure 2). However, TG, either alone or combined with EW, increased the expression of *Cldn2* and *Muc2*, particularly at the jejune level. Expression of *Cldn2* was not significantly augmented by administration of PL, while that of *Muc2* was enhanced in mice given PL or the mixture EW:PL as compared with mice given PBS, although in the jejunum it was lower than in those fed TG (Figure 2).

On the other hand, EL, as well as TG and PL, although to a lesser extent, increased the expression of *Cd1d1* in the duodenum and jejunum (Figure 2). *Cd1d1* codes for CD1d, a lipid-binding molecule on the surface of IECs and DCs that presents lipid antigens to invariant natural killer T 2 (iNKT2) cells, leading to the release of IL-4, which promotes the differentiation of Th2 cells and allergy expansion [18]. Noteworthily, the mixture of EW with EL, TG, or PL also stimulated *Cd1d1* expression, while EW alone did not (Figure 2).

**Figure 3:**
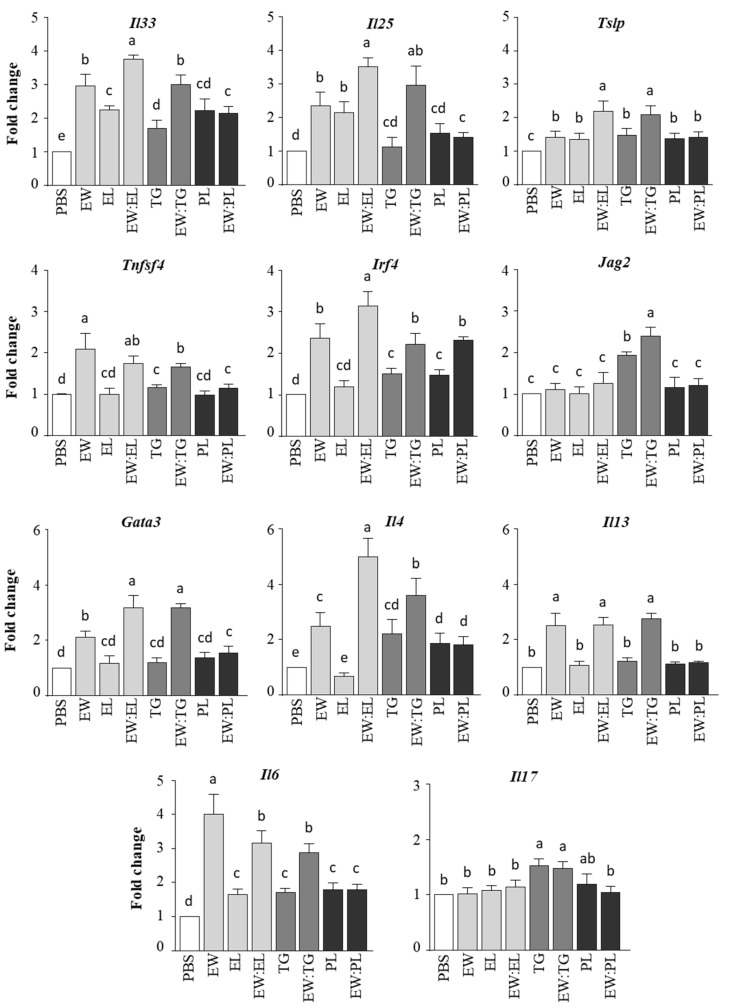
Relative gene expression of *Il33, Il25, Tslp*, *Tnfsf4, Irf4, Jag2, Gata3, Il4, Il13, Il6*, and *Il17*, determined in the duodenum of mice intragastrically administered PBS, egg white (EW, 50 mg), egg lipids (EL, 60 mg), a triacylglyceride-enriched fraction (60 mg, TG), a phospholipid-enriched fraction (60 mg, PL), and their mixtures (50 mg:60 mg) with EW (respectively EW:EL, EW:TG, EW:PL) for 6 consecutive days. Gene expression was normalized to the reference gene *Actb* and compared with mice administered PBS. Data are expressed as means ± SEM (*n* = 6). Different letters indicate statistically significant differences (*p* < 0.05) among different mouse groups.

As expected, in view of previous findings showing that the joint administration of EW and EY activated the expression of genes that code for epithelium-associated cytokines that promote Th2 responses (IL-33, IL-25, and TSLP) [8], we found that *Il33*, *Il25*, and *Tslp* were upregulated in the duodenum of mice given the mixture EW:EL over those given EW or EL alone (Figure 3). The study of the separate effect of the main EL components, TG and PL, showed that the mixture EW:TG exerted a more pronounced influence on the intestinal expression of *Il33*, *Il25*, and *Tslp* than the mixture EW:PL. EW:TG enhanced duodenal *Tslp* expression over EW alone, but not that of IL-33 and IL-25.

IL-33, IL-25, and TSLP promote on DCs the expression of genes that drive Th2 differentiation [19]. Therefore, we studied, in the duodenum of mice administered EW and its mixture with EL, TG, or PL, the regulation of DC Th2-skewing factors, such as *Tnfsf4* (encoding OX40L [20])*, Irf4* (encoding interferon regulatory factor 4 (IRF4) [21]), and *Jag2* (encoding the Notch ligand Jagged 2 [22]). EW upregulated *Tnfsf4* and *Irf4*, and coadministration of EL promoted *Irf4* expression (Figure 3). However, while neither TG nor PL enhanced the effect of EW on *Irf4*, EW:TG exerted a significantly higher influence than EW on the expression of *Jag2* (Figure 3).

Interestingly, the Th2 master transcription factor *Gata3* and the Th2 cytokine *Il4* were overexpressed in the duodenum of mice given EW:EL and EW:TG as compared with those exclusively fed EW. Furthermore, EW:EL and EW:TG led to high levels of *Il13* expression, comparable to those produced by the administration of EW alone, also upregulating *Il6*. Moreover, administration of EW:TG (as well as TG alone) increased duodenal expression of *Il17*. Both IL-6 and IL-17 are proinflammatory cytokines that contribute to the pathogenesis of several inflammatory diseases [23,24]. The immune responses induced by EW:EL and EW:TG in the jejunum were similar, with EW:TG exerting a significant influence on the expression of *Jag2*, *Il6*, and *Il17* (Supplementary Figure S2). On the other hand, in general terms, PL showed a negligible inducing effect on the regulation of genes involved in proallergenic (*Gata3, Il4, Il13*) or proinflammatory (*Il6, Il17*) responses in the small intestine of mice (Figure 3 and Supplementary Figure S2).

**Figure 4:**
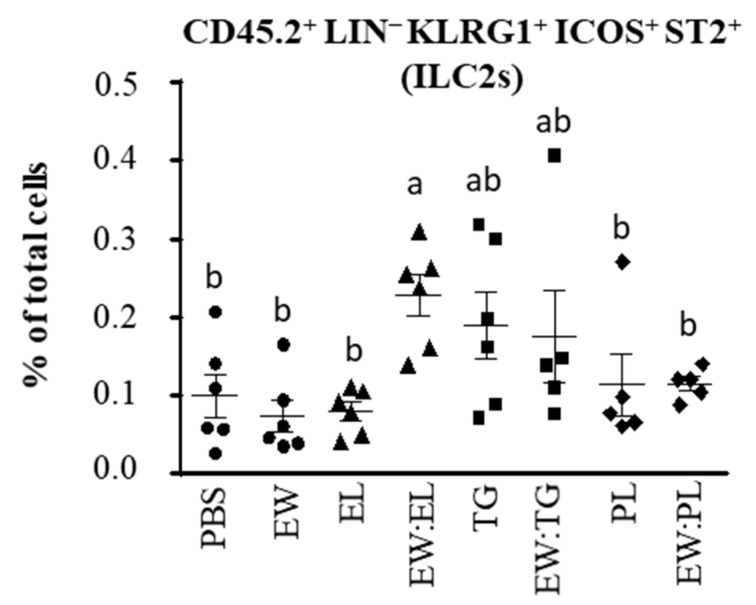
Group 2 innate lymphoid cells (ILC2s, defined as CD45.2^+^Lineage^−^ KLRG1^+^ICOS^+^ST2^+^) within the total cells in the lamina propria of mice intragastrically administered PBS, egg white (EW, 50 mg), egg lipids (EL, 60 mg), a triacylglyceride-enriched fraction (60 mg, TG), a phospholipid-enriched fraction (60 mg, PL) and their mixtures (50 mg:60 mg) with EW (respectively EW:EL, EW:TG, EW:PL) for 6 consecutive days. Data are expressed as means ± SEM (*n* = 6). Different letters indicate statistically significant differences (*p* < 0.05) among different mouse groups.

Another outcome of the epithelium Th2-biasing cytokines IL-33, IL-25, and TSLP is the expansion of ILC2s, which bear the transcription factor GATA3 and produce IL-4, IL-5, and IL-13 [25]. As expected in view of the above-explained results, the percentage of ILC2s was significantly increased in the lamina propria of mice administered EW:EL (Figure 4). TG and the mixture EW:TG also enhanced the level of ILC2s, although the difference did not reach statistical significance. Overall, these results indicate that EL, by virtue of its content in triacylglycerides, may exert effects in the small intestine, favoring the release of Th2-biasing cytokines that instruct DCs to promote Th2 responses and stimulate the expansion of ILC2s that work as an extra source of polarizing cytokines, altogether inducing and reinforcing Th2 immunity, which is critical in allergic sensitization.

### 3.2. Egg Yolk Phospholipids Induce Th2 Skewing in Lymphoid Intestinal Tissues

**Figure 5:**
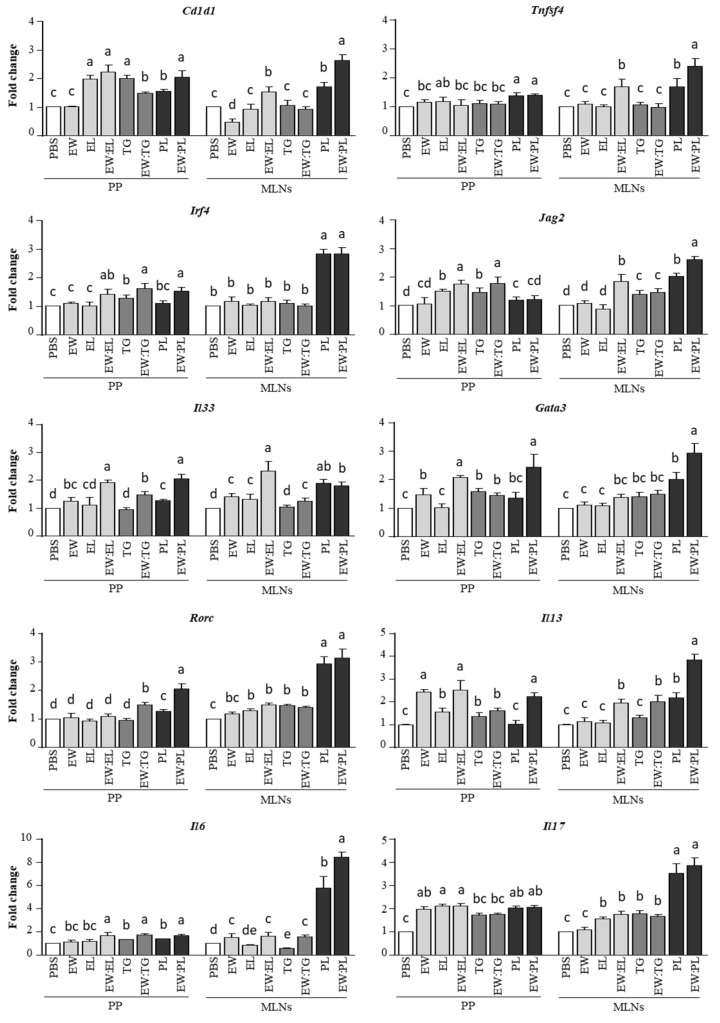
Relative gene expression of *Cd1d*, *Tnfsf4, Irf4, Jag2, Il33, Gata3, Rorc, Il13, Il6*, and *Il17*, determined in the Peyer’s patches (PP) and mesenteric lymph nodes (MLN) of mice intragastrically administered PBS, egg white (EW, 50 mg), egg lipids (EL, 60 mg), a triacylglyceride-enriched fraction (60 mg, TG), a phospholipid-enriched fraction (60 mg, PL), and their mixtures (50 mg:60 mg) with EW (respectively EW:EL, EW:TG, EW:PL) for 6 consecutive days. Gene expression was normalized to the reference gene *Actb* and compared with mice administered PBS. Data are expressed as means ± SEM (*n* = 6). Different letters indicate statistically significant differences (*p* < 0.05) among different mouse groups.

The assessment of the regulation of Th2-driving factors by DCs in the PPs revealed that coadministration of EL, TG, and PL stimulated the expression of *Cd1d1* and *Irf4*, as compared with the administration of EW alone (Figure 5). EL and TG also promoted the expression of *Jag2* induced by EW, and PL, as well as EW:PL, enhanced *Tnfsf4* expression (Figure 5). Interestingly, EW:EL and EW:PL upregulated *Il33*, whose expression in DCs and subsequent IL-33 production (induced by intestinal IL-33, as well as by certain lipids) is known to enhance Th2 responses [26]. In fact, the mixtures EW:EL and EW:PL upregulated *Gata3* over EW, with the combination EW:PL yielding the highest expression of *Rorc* (encoding RORγt, the key transcription factor of Th17 cells). As a result, and unlike the situation in the intestine, *Il13* was overexpressed in the PPs of mice given EW, EW:EL, and EW:PL, and a similar situation was observed in the case of *Il17* (also enhanced by EL and PL alone), although *Il6* was similarly overexpressed in the animals administered EW:EL, EW:TG, and EW:PL (Figure 5).

**Figure 6:**
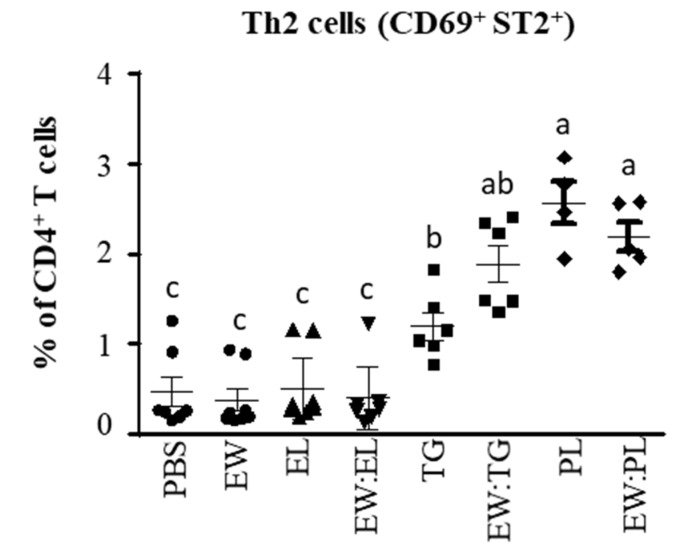
Activated (CD69^+^) ST2^+^ cells within CD4^+^ T cells in the MLNs of mice intragastrically administered PBS, egg white (EW, 50 mg), egg lipids (EL, 60 mg), a triacylglyceride-enriched fraction (60 mg, TG), a phospholipid-enriched fraction (60 mg, PL), and their mixtures (50 mg:60 mg) with EW (respectively EW:EL, EW:TG, EW:PL) for 6 consecutive days. Data are expressed as means ± SEM (*n* = 6). Different letters indicate statistically significant differences (*p* < 0.05) among different mouse groups.

Noteworthily, the effect of the PL fraction was remarkable in the MLNs, where the mixture EW:PL distinctively enhanced the expression of *Cd1d1*, *Tnfsf4*, *Irf4*, *Jag2*, *Gata3*, *Rorc, Il13, Il6*, and *IL17* to an extent comparable to or higher than EW:EL, also increasing that of *Il33*, while TG combined with EW exerted a much lower effect (Figure 4). Accordingly, PL and EW:PL promoted high numbers of activated (CD69^+^) Th2 cells (CD4^+^ cells bearing the IL-33 receptor ST2 [27]) in the MLNs (Figure 6). These results thus show that the administration of the combination of EW and PL increased the Th2-priming ability of DCs, induced the expansion of Th2 cells and associated cytokines, and stimulated proinflammatory Th17 responses in intestine-associated lymph nodes.

### 3.3. Egg Yolk Phospholipids Reduce the Solubility of Egg White Proteins in a Simulated Duodenal Medium

We next incubated EW and its mixtures with EL, TG, and PL in a simulated duodenal digestion medium that resembles a state intermediate between a fasted state (characterized by a low pH and a low concentration of bile salts) and a fed state (with higher pH and concentration of bile salts) [28], without digestive enzymes. Solubility of EW was maintained in the presence of EL, but the lipid fractions, and particularly PL, partially induced precipitation of EW proteins (Supplementary Figure S3). This observation suggests that, although bile salts exert a solubilizing effect, removing proteins adsorbed to emulsions and favoring their dispersion in aqueous phases [29], high concentrations of phospholipids may promote their precipitation, which could impair hydrolysis by pancreatic enzymes and absorption in the intestinal tract.

## 4. Discussion

There is evidence that lipids can shape both innate and adaptive immunity, ultimately modifying the immunostimulating properties of proteins [6]. In this work, a model in vivo system was used to elucidate the role of triglycerides and phospholipids in the early events of sensitization to egg proteins. An acute administration protocol was followed to avoid the strong tendency of mice to develop tolerance. On the other hand, and although eggs are usually ingested after being subjected to heat treatments of different intensities, we deliberately avoided heating in this study in order to assess the potential adjuvant activity of the main lipid fractions without the interference of other effects derived from denaturation and possible aggregation of proteins and their interaction with lipids. Our results highlight that the egg lipid fraction is responsible for the Th2 adjuvant effect of egg yolk and point at a different effect of triacylglycerides and phospholipids on the gastrointestinal response.

Lipids might change the susceptibility of proteins to gastrointestinal digestion and facilitate their intestinal transport through lipid-mediated uptake or disruption of the intestinal barrier [6]. In this respect, however, it was previously shown that egg yolk does not exert a major influence on the in vitro digestibility of egg white proteins [30]. Furthermore, whereas dietary fat was found to increase intestinal permeability by reducing the expression of proteins that downregulate the opening of tight junctions [31], the observation that the joint administration of EW and EL withdrew the enhancing effects of EW on the expression of *Muc2* and *Cldn2* indicates that EL did not favor the epithelial passage of EW proteins, in agreement with earlier studies from our group showing that egg yolk does not amplify allergic reactions to egg white in egg white-sensitized mice [7].

In contrast to EW:EL, EW:TG was shown to have a stimulatory effect on *Cldn2* expression in the duodenum and jejunum, which may have caused an increased permeability to ions and macromolecules [16]. The concomitant upregulation of *Muc2* points at the activation of tissue-repair mechanisms by the induction of mucus secretion [17]. Moreover, the enhanced expression of proinflammatory factors, such as *Il17* and *Il16*, detected particularly in the jejunum of mice given EW:TG, reinforces the concept that coadministration of TG, unlike that of PL, enhanced the intestinal absorption of EW proteins. It is recognized that dietary factors that facilitate allergen passage by weakening the intestinal barrier and triggering inflammation play a role in the development of food allergy [32]. In fact, the strong Th2 adjuvant cholera toxin (CT), commonly used to induce food allergy in mouse models, enhances the expression of *Cldn2*, *Muc2*, *Il17*, and *Il6* in the small intestine when administered with food proteins to produce sensitization [33]. In line with those findings, administration of EW:TG promoted, to a higher extent than EW:PL, the expression of genes coding for Th2-promoting epithelial cytokines (*Il33*, *Il25*, and *Tslp*), Th2-skewing DC factors (*Tnfsf4* and *Jag2*), and Th2 markers (*Gata3, Il4* and *Il13*), although, in general terms, the highest responses in the duodenum and jejunum corresponded to mice given EW:EL, which also experienced high levels of ILC2s in the lamina propria.

Whereas the aforementioned results indicate that TG altered the intestinal barrier function and worked as a local Th2 stimulus, comparatively, PL exerted a higher influence in the PP and MLN, which work as overlapping inductive sites in tolerance and sensitization. Indeed, the adjuvant CT, orally fed to mice, generates allergy in vivo by inducing upregulation of DC genes that prime for Th2 responses, such as *Irf4, Tnfsf4*, and *Jagged2*, in the PPs and MLNs [8,19,34]. In the PPs, EW:PL showed an effect higher than EW alone, and similar to that of EW:EL, in the expression of *Tnfsf4, Il33*, and *Gata3*, while considerably stimulating the expression of *Rorc.* Noteworthily, in the MLNs, EW:PL distinctively activated DC genes that bias T cells towards a proallergenic or inflammatory response, also enhancing the expression of genes that code for transcription factors and cytokines typical of Th2 and Th17 cells. These results suggest that PL aided antigen delivery to PPs to stimulate T cells, which then pass to the MLNs where the immune response is amplified. Indeed, absorption through M cells, able to transport luminal antigens to the underlying immune cells, is believed to increase their ability to induce Th2 immunity as compared with epithelial absorption [35].

It is known that long-chain triacylglycerides (with more than 12 C atoms, such as those predominant in EL) are digested by pancreatic lipase in the upper segment of the jejunum, finally releasing glycerol and long-chain free fatty acids that are taken up from the intestinal lumen and used for the biosynthesis of complex lipids. Inside the IECs, these are further assembled and secreted in chylomicrons, which transport them via the lymphatic vessels and systemic circulation to different tissues [36]. Wang et al. [9] found that administration to mice of allergens together with long-chain triacylglycerides enhances absorption through IECs and systemic dissemination via chylomicron formation, eventually promoting oral tolerance. Conversely, coadministration of shorter-chain triacylglycerides or other lipids that are preferentially uptaken through PPs favors subsequent transport of proteins to the draining MLNs and increases their immunogenicity [10].

It should be noted that, similarly to long-chain triacylglycerides, orally ingested phospholipids are almost completely absorbed in the upper small intestine, taken up by IECs, and secreted in chylomicrons. In the intestinal lumen, their digestion is carried out primarily by pancreatic phospholipase A2 (pPLA2) and other lipases to yield free fatty acids and lysophospholipids, which are removed from the water–oil interface and incorporated into mixed micelles that form spontaneously with the aid of bile salts and also contain triacylglycerides, phospholipids, monoacylglycerides, and cholesterol [37]. However, an excess of phospholipids impedes their normal pPLA2-driven hydrolysis, interfering with their efficient mucosal uptake and also hindering cholesterol uptake. Alternatively, high concentrations of phospholipids could affect the physicochemical properties of mixed micelles leading to poor absorption [36]. These two mechanisms, which have been postulated to describe how the amount and type of phospholipids restrict cholesterol uptake and eventually lead to inhibition of intestinal cholesterol absorption, could explain the observation that the egg PL fraction was preferentially taken through PPs over IECs, increasing the immunogenicity of the accompanying EW. Indeed, in vitro experiments revealed that solubility of EW in simulated duodenal fluid was distinctly compromised when it was mixed with PL in the same proportion as that given to mice. It should be noted that eggs, by virtue of their high content of phospholipids, have consistently been reported to reduce cholesterol absorption, increasing its fecal output [38]. Therefore, impaired epithelial absorption of phospholipids, leading to delivery through PPs, could also be expected when ingested as part of a whole egg, accounting for its Th2-promoting potential found in this study, as well as in previous reports.

Indeed, EL triggered a strong immune response, likely because it may favor antigen both passage through the IECs, which ultimately reaches the MLNs carried by DCs migrating from the lamina propria, and direct transport through M cells to the underlying immune cells of the PPs and, via efferent lymphatic vessels, to the MLNs. In addition, EL specifically induced *Cd1d1* expression in the intestine, although in the MLNs, the highest *Cd1d1* expression corresponded to mice administered PL. Recognition of lipids and lipid-binding proteins presented through CD1d by iNKTs triggers the upregulation of costimulatory molecules and the secretion of cytokines, helping iNKTs to act as effector cells and amplifiers of Th2 responses [39].

In conclusion, our results show that the main egg lipid components, triacylglycerides and phospholipids, differently influence the bioavailability of egg proteins, changing their immunostimulating properties, particularly their ability to stimulate Th2 responses, which may have a direct impact on the development of allergy.

## Data Availability

Not applicable.

## Supplementary Materials

The following are available online at https://www.mdpi.com/article/10.3390/nu13103301/s1, Figure S1: Chromatographic profile (a) and percentage of lipid classes (b) of EL, TG, and PL fractions determined by HPLC-ELSD; Figure S2: Relative gene expression of *Il33, Il25, Tslp*, *Tnfsf4, Irf4, Jag2, Gata3, Il4, Il13, Il6*, and *Il17*, determined in the jejunum of mice intragastrically administered PBS, EW, EL, EW:EL, EW:TG, and EW:PL for 6 consecutive days; Figure S3: SDS-PAGE of EW and its mixtures with EL, PL, and TG in a simulated duodenal digestion medium; Table S1: Antibodies used for the analysis of T-cell and ILC2 subsets by flow cytometry.
