# Supplementary material for: Triacylglycerides and Phospholipids from Egg Yolk Differently Influence the Immunostimulating Properties of Egg White Proteins
Source: Nutrients. 2021 Sep 22;13(10):3301. doi: 10.3390/nu13103301 (PMC8539141; doi:10.3390/nu13103301)
Supplement: Supplementary file 1 [file nutrients-13-03301-s001.zip › nutrients-1351885-supplementary.pdf]

Supplementary Table S1. Antibodies used for the analysis of T cell and ILC2 subsets by flow cytometry (all from eBioscience, San Diego, CA, USA).

Population	Antibody		Clone	Fluorophore
	CD16/CD32		2.4G2	
$CD4^+$ T cells	CD4		GK1.5	Alexa Fluor 700
	CD69		H1.2F3	PerCP-Cy5.5
	ST2 (IL-33 receptor)		RMST2-2	PE
ILC2	CD45.2		104	PE-Cy7
	Hematopoietic lineage	CD3	17A2	FITC
		CD45R (B220)	RA3-6B2	
		CD11b	(M1/70)	
		TER-119	TER-119	
		Ly-G6 (Gr-1)	RB6-8C5	
		CD19	MB19-1	
	ST2 (IL-33 receptor)		RMST2-2	PE
	KLRG1		2F1	APC eFluor 780
	CD278 (ICOS)		C398.4A	APC

a)

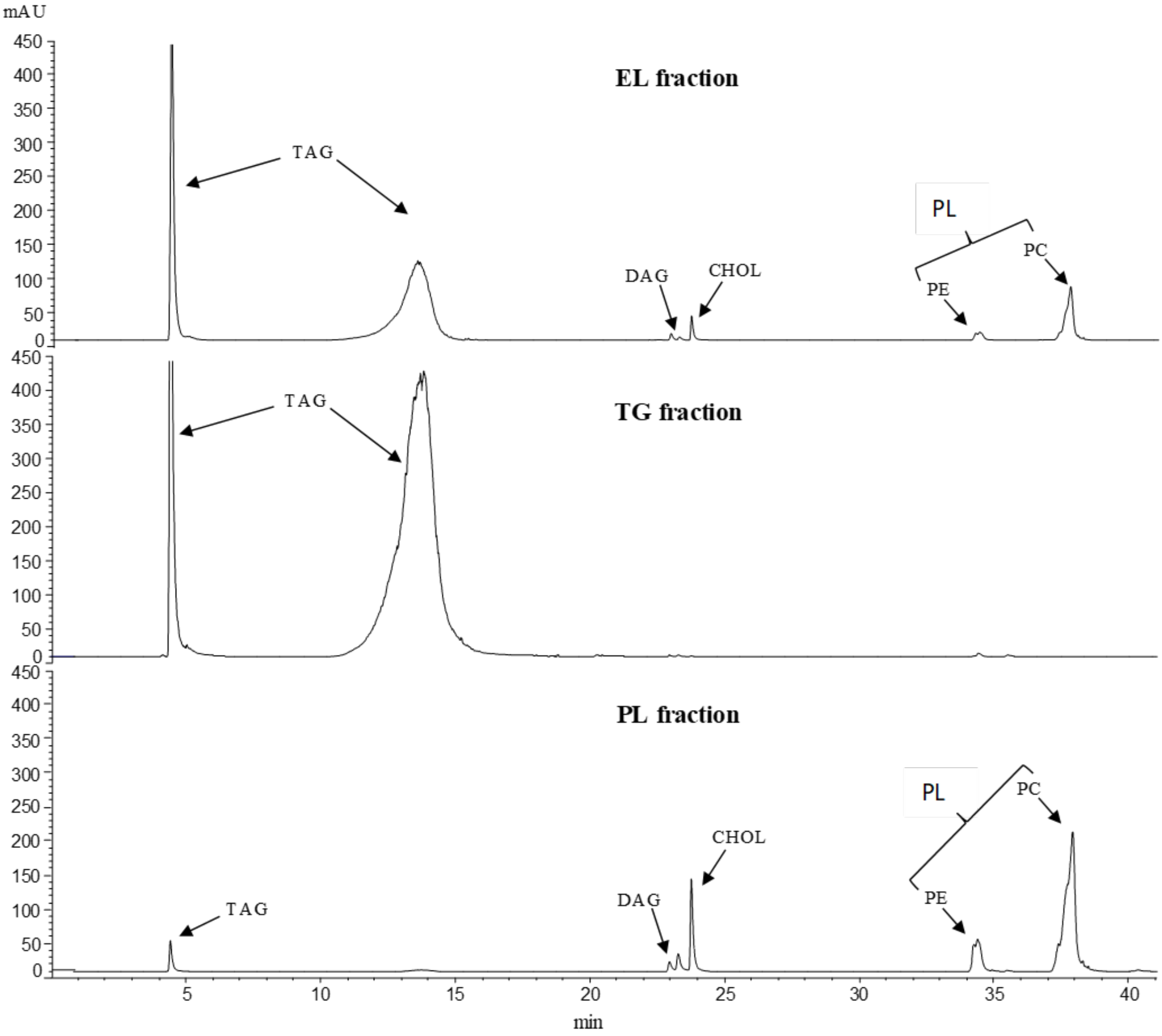

b)

Lipid classes	EL fraction	TG fraction	PL fraction
TAG	86.30 %	99.78 %	6.47 %
DAG	0.69 %	0.05 %	3.99 %
CHOL	1.52 %	0.01 %	11.75 %
PL	11.47 %	0.14 %	77.79 %

Supplementary Figure S1. Chromatographic profile (a) and percentage of lipid classes (b) of EL, TG, and PL fractions determined by HPLC-ELSD. (TAG, triacylglycerides; DAG, diacylglycerides; CHOL, cholesterol; PL, phospholipids; PE, phosphatidylethanolamine; PC, phosphatidylcholine)

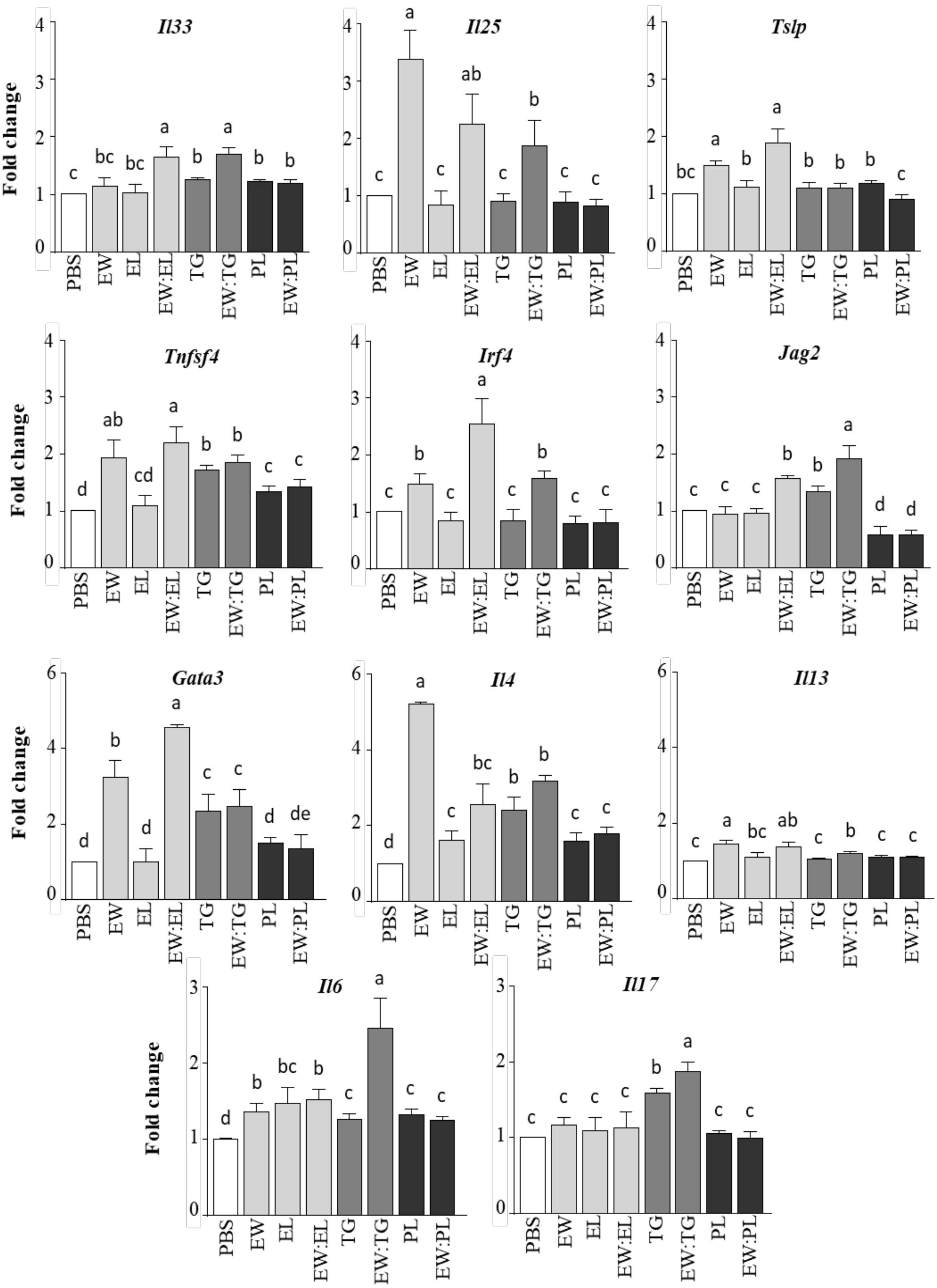

Supplementary Figure S2. Relative gene expression of *Il33, Il25, Tslp*, *Tnfsf4, Irf4, Jag2, Gata3, Il4, Il13, Il6,* and *Il17*, determined in the jejunum of mice administered intragastrically:

PBS, EW, EL, EW:EL, EW:TG, EW:PL, for 6 consecutive days. Gene expression was normalized to the reference gene *Actb* and compared with mice administered PBS. Data are expressed as means ± SEM (n=6). Different letters indicate statistically significant differences (p<0.05) among different mouse groups.

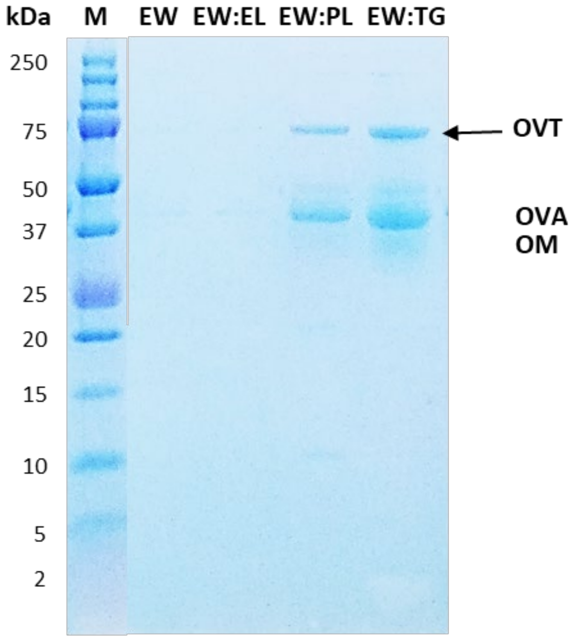

Supplementary Figure S3. SDS-PAGE of EW and its mixtures with EL, PL and TG in a simulated duodenal digestion medium, M: molecular mass marker, lane 1: EW; lane 2, EW:EL; lane 3, EW:PL and line 4, EW:TG.
